# The Stability of Problem Behavior Across the Preschool Years: An Empirical Approach in the General Population

**DOI:** 10.1007/s10802-015-9993-y

**Published:** 2015-04-02

**Authors:** Maartje Basten, Henning Tiemeier, Robert R. Althoff, Rens van de Schoot, Vincent W. V. Jaddoe, Albert Hofman, James J. Hudziak, Frank C. Verhulst, Jan van der Ende

**Affiliations:** 1 Department of Child and Adolescent Psychiatry/Psychology, Erasmus Medical Center, P.O. Box 2060, 3000 CB Rotterdam, The Netherlands; 2 The Generation R Study Group, Erasmus Medical Center, Rotterdam, The Netherlands; 3 Department of Epidemiology, Erasmus Medical Center, Rotterdam, The Netherlands; 4 Department of Psychiatry, Erasmus Medical Center, Rotterdam, The Netherlands; 5 Vermont Center for Children, Youth, and Families, The University of Vermont, Burlington, VT USA; 6 Department of Methods and Statistics, Utrecht University, Utrecht, The Netherlands; 7 Optentia Research Program, Faculty of Humanities, North-West University, Vanderbijlpark, South Africa; 8 Department of Pediatrics, Erasmus Medical Center, Rotterdam, The Netherlands

**Keywords:** Preschool internalizing and externalizing problems, Stability, Longitudinal study, Co-occurrence, Dysregulation, Latent transition analysis

## Abstract

This study examined the stability of internalizing and externalizing problems from age 1.5 to 6 years, while taking into account developmental changes in the presentation of problems. The study comprised a population-based cohort of 7,206 children (50.4 % boys). At ages 1.5, 3, and 6 years, mothers reported on problem behavior using the Child Behavior Checklist/1.5-5 (CBCL/1.5-5). At each age we performed latent profile analysis on the CBCL/1.5-5 scales. Latent transition analysis (LTA) was applied to study the stability of problem behavior. Profiles of problem behavior varied across ages. At each age, 82–87 % of the children did not have problems whereas approximately 2 % showed a profile of co-occurring internalizing and externalizing problems. This profile was more severe (with higher scores) at 6 years than at earlier ages. A predominantly internalizing profile only emerged at 6 years, while a profile with externalizing problems and emotional reactivity was present at each age. LTA showed that, based on profiles at 1.5 and 3 years, it was difficult to predict the type of profile at 6 years. Children with a profile of co-occurring internalizing and externalizing problems early in life were most likely to show problem behavior at 6 years. This study shows that the presentation of problem behavior changes across the preschool period and that heterotypic continuity of problems is very common among preschoolers. Children with co-occurring internalizing and externalizing problems were most likely to show persisting problems. The use of evidence-based treatment for these young children may prevent psychiatric problems across the life course.

**Electronic supplementary material:**

The online version of this article (doi:10.1007/s10802-015-9993-y) contains supplementary material, which is available to authorized users.

It has been recognized that emotional and behavioral problems start at a young age (Egger and Angold 2006). This recognition has led to an increase in studies on preschool emotional and behavioral problems and their stability over time. Studying the stability of problems from a young age onwards will increase our understanding of the development of psychopathology and will help in early identification of children at risk for psychopathology later in life. Defining emotional and behavioral problems in preschoolers is challenging, as behaviors that are considered “problematic” at older ages are part of normative development in early childhood (Carter et al. 2004). Developmental changes in the presentation of internalizing, externalizing and the co-occurrence of internalizing and externalizing problems need to be considered in studying the continuity of problems among preschool children.

There is clear evidence that the core distinctions of internalizing problems (such as anxiety and depression) and externalizing problems (such as aggression, hyperactivity, and impulsivity) observed in older children and adolescents are also observed in preschoolers (Achenbach and Rescorla 2000; Carter et al. 2003; Sterba et al. 2007a). Several studies have examined the persistence of internalizing and externalizing problems by examining associations between levels of problems measured at different ages. Substantial stability has been found (Campbell 1995; Fischer et al. 1984; Keenan et al. 1998; Mathiesen and Sanson 2000; Mesman et al. 2001; Mian et al. 2011). For example, Mesman et al. (2001) found that internalizing and externalizing problems at ages 2–3 years predicted the same type of problems at ages 10–11 years. These results show homotypic stability, which is defined as the continuity of some phenomenon over time in a form that changes relatively little (Angold et al. 1999). Also heterotypic stability has been found, which is defined as manifestations of different forms over time (Angold et al. 1999). For example, Mesman et al. (2001) found that preschool levels of externalizing problems predicted higher levels of internalizing problems at ages 10-11 years.

In addition to association studies, several groups have examined stability using latent class growth analyses – a technique that allows for the study of unobserved (latent) patterns of behavior over time. Children with patterns of persistent internalizing problems or persistent externalizing problems have been identified (Fanti and Henrich 2010; Sterba et al. 2007b; Tremblay et al. 2004). Again, these studies tend to concentrate on a single domain over time. Fanti and Henrich (2010), however, identified groups of children with persistent internalizing problems, persistent externalizing problems, and persistent co-occurring internalizing and externalizing problems over time.

Association studies and latent class growth models have been highly informative on our understanding of the stability of problems across the preschool period and beyond. However, they give limited information at the level of the individual child. Identification of preschool children who are most likely to have persisting problems would be most useful for prevention and intervention. Therefore persistence has also been studied categorically using symptom cut-points or diagnostic classifications (Briggs-Gowan et al. 2006; Bufferd et al. 2012; Campbell 1995; Keenan et al. 1998; Lavigne et al. 1998; Luby et al. 2009; Mathiesen and Sanson 2000; Speltz et al. 1999). For example, Briggs-Gowan et al. (2006) found that 50 % of 12-to-40 month olds with high levels of parent reported externalizing problems, showed high levels of externalizing problems 1 year later. Similarly, children with internalizing problems had a 38 % chance to have internalizing problems after 1 year. Interestingly, children with co-occurring internalizing and externalizing problems were most likely to have persistent problems. Bufferd et al. (2012) showed persistence of attention deficit hyperactivity disorder, oppositional defiant disorder, and anxiety disorders from age 3 to age 6 years. They also found continuity across diagnoses: for example children meeting the criteria for an anxiety disorder at age 3 years were more likely to meet the criteria for depression and oppositional defiant disorder at age 6 years.

A major challenge for studies on the categorical stability of preschool problems is to distinguish deviant behavior from normal development (Carter et al. 2004). Longitudinal studies have shown that overall levels of externalizing problems decrease from ages 2 to 6 years (Fanti and Henrich 2010; Gilliom and Shaw 2004). This is explained by the recognition that externalizing behaviors such as oppositional behavior and temper tantrums are part of normal behavior when children are younger, and in many children these behaviors decrease over time. Some types of internalizing problems are also considered ‘normal’ at younger ages, such as separation anxiety. However, although some studies found a decreasing pattern of internalizing problems across the preschool period (Carter et al. 2010; Sterba et al. 2007b), others found an increase (Colder et al. 2002; Gilliom and Shaw 2004). Little is known regarding the development of co-occurring internalizing and externalizing problems. One theory suggests that co-occurrence decreases with age as psychopathology becomes more differentiated (Nottelmann and Jensen 1995), while studies on DSM disorders in preschoolers have found increases in the prevalence of comorbidity over time (Egger and Angold 2006; Lavigne et al. 1998).

Most studies on the stability of problems in preschoolers have applied the same criteria to define problem behavior at each age. By using the same criteria or cut-points across different ages on the same instrument, the deviance from typical development is not taken into account appropriately. An alternative is to use age-specific cut-points on scales of problem behavior. For example, Briggs-Gowan et al. (2006) used standardized scores within 6-month age bands and set cut-points at the 90th percentile, which results in 10 % of the children having problems at each age. With the use of these age-specific cut-points it is assumed that the prevalence of problems is equal across ages, which is questionable. A third possibility is using different age-specific instruments to assess problem behavior (e.g., Keenan et al. 1998). A disadvantage of this approach however is that differences between instruments limit the comparability over time.

An empirical way to study the individual stability of problem behavior, while taking into account developmental changes in the presentation of problem behavior, is by using latent transition analysis (LTA; Collins and Lanza 2010). Latent transition analysis is a longitudinal extension of cross-sectional person centered methods such as latent class analysis (LCA) and latent profile analysis (LPA). LCA and LPA are methods to empirically identify homogeneous groups of individuals with similar profiles of problem behavior on a set of categorical variables (LCA) or dimensions (LPA). Changes in the presentation of problem behavior can be identified by comparing latent profiles derived at different ages. Subsequently, in latent transition analysis, children’s transitions in profiles across ages can be examined. These methods have important advantages. In person-centered methods such as LPA and LTA groupings are empirically based and not based on cut-points, which are arbitrary and less developmentally sensitive. However, in finding the optimum solution on the number of profiles both statistical information and substantive reasons have to be considered. Additionally, profiles can be estimated across the range of internalizing and externalizing problems. In this way, the stability of co-occurring internalizing and externalizing problems can be studied. Thus, LTA, if used on an instrument that is consistent over the developmental period in question, allows for the examination of the stability of internalizing and externalizing problems without the confounds that have plagued categorical, cutpoint-based research to date.

One study has examined the stability of internalizing and externalizing problems in preschoolers using LTA to examine the effects of a family intervention (Connell et al. 2008). They examined children at 2, 3, and 4 years of age. At each age they identified four classes: ‘externalizing only’, ‘internalizing only’, ‘comorbid internalizing and externalizing’, and ‘normative’. These classes were assumed to be similar across ages, but this was not tested. Children in the ‘externalizing only’, ‘internalizing only’, and ‘comorbid internalizing and externalizing’ classes, who did not receive treatment, were likely to be in the same class after 1 year (transition probabilities ranged from 0.53 to 0.86; Connell et al. 2008). The children in the study of Connell et al. (2008) were selected to be at particularly high risk and from lower SES families, which may have influenced the identified classes and the stability of class membership over time. Therefore it is unknown if these same results would be seen in the general population.

The current study was designed to examine profiles of internalizing and externalizing problems throughout the preschool period and examine the stability of problems over time. In contrast to many previous studies that were based on high risk samples, this study was performed in a large general-population sample. Examining stability in the general population may extend our knowledge of child development and may be more informative for the development of prevention strategies in the general population. Our first aim was to evaluate the architecture and prevalence of profiles of internalizing and externalizing problems at ages 1.5, 3 and 6 years using latent profile analysis. Based on our previous findings using LPA to empirically derive profiles of emotional and behavioral problems in 6 year old children (Basten et al. 2013) we hypothesized to find profiles with predominantly internalizing problems and profiles with predominantly externalizing problems to exist at 1.5 years of age. We further hypothesized that a profile with co-occurring internalizing and externalizing problems would be found at each age. Moreover, because levels of externalizing problems are higher in younger than in older children (Fanti and Henrich 2010; Gilliom and Shaw 2004), we hypothesized profiles characterized by externalizing problems to be more prevalent at the beginning of the preschool period than at a later age.

Our second aim was to study the stability of these profiles by examining children’s transitions in profiles from 1.5 and 3 to 6 years of age using LTA. Based on earlier findings on the stability of co-occurring internalizing and externalizing problems (Briggs-Gowan et al. 2006) we hypothesized that children with a profile of co-occurring internalizing and externalizing problems were more likely than children with problems on a single domain to have persisting problems.

Finally we also studied gender differences in the stability of problems. Previous studies examining categorical stability across the preschool period found few gender differences (Briggs-Gowan et al. 2006; Bufferd et al. 2012; Lavigne et al. 1998), but externalizing problems have been demonstrated to become more prevalent in boys than girls during the preschool period (Hay 2007; Rutter et al. 2003). Therefore we hypothesized that externalizing problems would be more persistent in boys than in girls.

Determining profiles of co-occurring externalizing and internalizing problems in preschool children, their stability over time, and their relations to gender may allow for future identification and intervention for the most affected children later in life.

## Method

### Setting and Population

This study was embedded in the Generation R Study, a multi-ethnic population-based cohort from fetal life onwards in Rotterdam, The Netherlands. The Generation R Study has been described previously (Jaddoe et al. 2012; Tiemeier et al. 2012). Briefly, all pregnant women living in Rotterdam, with an expected delivery date between April 2002 and January 2006 were invited to participate. The study was approved by the Medical Ethics Committee of the Erasmus Medical Center, Rotterdam. Written informed consent was obtained from all adult participants and anonymity was guaranteed. At birth, 9,749 children participated in the study (participation rate 61 %). Primary caregivers reported on child’s problem behavior at ages 1.5 years (*n* = 5,184), 3 years (*n* = 4,928), and 5-to-7 years (*n* = 6,131). In the present study we included 7,206 children for which at least one measurement was available (follow-up rate 74 %). At age 3 years partner report on the child’s problem behavior was also available in 4,010 children. At 5-to-7 years, the majority were 5 years old (58 %) and some children were 6 (38 %), or 7 (4 %) years old. Because the mean age was 6.0 (SD = 0.4) this wave was hereafter referred to as ‘age 6’. Table 1 presents sample characteristics.

**Table 1:** Sample Characteristics

	*N* = 7,206
Gender %	
Girls	49.6
Boys	50.4
Age at each wave, years, mean (SD)	
Age 1.5	1.5 (0.1)
Age 3	3.1 (0.1)
Age 6	6.0 (0.4)
Child ethnicity %	
Dutch	58.8
Other Western	8.9
Non-Western	31.0
Missing	1.3
Maternal education^a^ %	
High	46.2
Medium/low	46.4
Missing	7.5
Family income (net per month) ^b^ %	
>€2,000	52.5
<€2,000	25.3
Missing	22.1

^a^ Maternal education level was defined as highest education finished. Education categories represent medium/low: primary school or lower vocational education or intermediate vocational education; high: higher vocational education or university. ^b^ Family income categories were defined by using a cut-off at €2,000 which is equal to modal family income

### Child Behavior Checklist

We assessed problem behavior using the Child Behavior Checklist for ages 1.5 to 5 (CBCL/1.5-5; Achenbach and Rescorla 2000). This version was also chosen at age 6 for continuity. At all ages the CBCL/1.5-5 was completed by the primary caregiver, which were mostly mothers (age 1.5 95 % mothers; age 3 92.5 % mothers; age 6 92.6 % mothers). At age 3, the primary caregivers’ partners, most of whom were fathers (89.5 %) reported on problem behavior of the child. Hereafter, primary caregivers are referred to as mothers and primary caregivers’ partners are referred to as fathers. The CBCL/1.5-5 consists of 100 problem items. Based on the behavior of the child in the preceding 2 months, the caregiver rated each item as 0 for not true, one for somewhat or sometimes true, and two for very true or often true. We used the empirically derived syndrome scales Emotionally Reactive, Anxious/Depressed, Somatic Complaints and Withdrawn, comprising the internalizing domain, and Attention Problems and Aggressive Behavior, comprising the externalizing domain. Good reliability and validity have been reported for the CBCL/1.5-5 (Achenbach and Rescorla 2000) and the scales were found to be generalizable across 23 societies, including The Netherlands (Ivanova et al. 2010). Ranges of Cronbach’s alphas for the scales at each age are 0.49–0.86 at age 1.5, 0.51–0.86 at age 3, and 0.60–0.89 at age 6. At the third wave, Cronbach’s alphas for all scales were the same in 5 year-old children and in children older than 5, indicating that problems were also reliably measured in children older than 5.

### Socio-Economic Status (SES)

Family income and maternal education level were examined at enrollment. Family income was defined by the total net month income of the household and categorized as ‘< 2,000’ (below modal income), and ‘> €2,000’ (more than modal income). Maternal education level was defined as highest education finished and was classified into two categories: medium/low (primary school, lower vocational education or intermediate vocational education), and high (higher vocational education or university).

### Data Analysis

Our first aim was to examine the development of profiles of problem behavior during the preschool period. We examined profiles of problem behavior at ages 1.5 and 3 using latent profile analysis (LPA) on the CBCL/1.5-5 completed by mothers. LPA identifies homogeneous latent classes of individuals with similar profiles on a set of continuous variables. This procedure was similar to the LPA previously performed at age 6 (Basten et al. 2013). As indicators, we used T-scores based on the American norm data on the CBCL/1.5-5 which are also applicable to the Dutch population (Achenbach and Rescorla 2010). Using T-scores makes the indicators comparable across classes and age. Further, T-scores will help interpretation because they are commonly used in clinical practice. A maximum likelihood estimator robust for skewness was used. We used five criteria to determine the number of profiles: 1) Bayesian information criterion (BIC) with lower values indicating better fit, 2) Bootstrapped Likelihood-Ratio Test (BLRT) which tests whether an additional profile improves the model, 3) Entropy with values towards 1 indicating better classification, 4) Profiles should include at least 1 % of the participants, 5) An additional profile should differ considerably in severity or shape from the other profiles.

Next, we tested whether profiles were equal across ages. The concept that a latent variable has the same measurement characteristics over time or across groups is known as measurement invariance (Collins and Lanza 2010). We estimated four models: 1) a model where profiles were allowed to vary across ages, 2) a model with equal profiles across ages 1.5 and 3, 3) a model with equal profiles across ages 3 and 6, and 4) a model holding profiles equal across all ages. Differences between these models were assessed by comparing BIC values. We also tested whether the profiles were the same between parent informants. We used the data collected from mothers and fathers at age 3 for this test. The model fit was again assessed through BIC values.

For the interpretation of the profiles, T-scores around 65 and higher were considered high, T-scores around 60 were considered moderate (in line with mean T-scores of 57–62 that were found for children referred to a mental health institution (Achenbach and Rescorla 2000), and T-scores around 55 were considered to be mild problem scores. In comparison, a matched group of non-referred children from the general population had mean T-scores of 54 (Achenbach and Rescorla 2000).

Our second aim was to study the stability of problem behavior in children. Therefore we performed latent transition analysis (LTA; Collins and Lanza 2010; Lanza and Collins 2008; Meeus et al. 2011). LTA is a person-centered method to study the stability and change of profile membership over time. LTA estimates transition probabilities from a particular profile at time *t* to another profile at time *t* + 1. If profiles are not equal over time, the qualitative change in profiles should be taken into account for interpreting the transition probabilities (Collins and Lanza 2010).

To test our last hypotheses, we examined gender and SES differences in profile prevalence rates and transitions. At each age we assigned children to their most likely latent profile (justified if entropy is > 0.80; Clark and Muthen 2009). We performed multinomial logistic regression analysis to test if gender and SES variables were related to profile membership. Subsequently, we added gender and SES variables as covariates to the LTA model to obtain transition probabilities for boys and girls and for low SES and high SES groups.

To deal with missing values in LTA, full-information maximum likelihood was used. Moreover, models were estimated on the basis of all information available from both complete cases (53.2 %) and cases with 1 (27.9 %) or 2 (18.9 %) measurements. All analyses were performed in Mplus version 7 (Muthén and Muthén 1998–2012). To examine possible biases of this method, we repeated LTA in the complete cases.

### Non-Response Analysis

We compared prenatal child and maternal characteristics of the children included in the analysis (*n* = 7,206) with those excluded because of no CBCL/1.5-5 available (*n* = 2,543). Children of responding mothers were more likely to be Dutch (58.8 vs. 25.9 %, *χ* ^2^ = 1,761, df = 3, *p* < 0.001). Responding mothers were more likely to be higher educated (46.2 vs. 13.3 % higher education, *χ* ^2^ = 1,320, df = 3, *p* < 0.001) and to have a more than modal family income (> €2,000 net per month) during pregnancy (52.5 vs. 13.2 %, *χ* ^2^ = 1,464, df = 3, *p* < 0.001).

## Results

### Creating Profiles and Testing Measurement Invariance

At age 6, a four-profile solution was considered the best fitting model (Basten et al. 2013). At ages 1.5 and 3 the BIC and the BLRT indicated that five profiles resulted in better model fit than four profiles (fit indices are reported in Supplementary Table S1). However, at age 1.5 the fifth profile consisted of only two participants with extreme scores. These measurements were considered outliers and were removed. After removal, we estimated a model with five profiles, but this time, the fifth profile had a shape similar to another profile, with only a slight difference in severity. Therefore, we chose a four-profile solution. At age 3, a model with five profiles was not the best option, as one of the profiles had a prevalence of less than 1 %. Thus, as we did at age 1.5, we decided to use a four-profile solution at age 3.

The measurement invariance test showed that a model with varying profiles across all ages had a lower BIC (502,960) than models with equal profiles across ages 1.5 and 3 (BIC = 503,398), across ages 3 and 6 (BIC = 503,964), or across all ages (BIC = 505,547). These results did not support the assumption of measurement invariance, indicating that profiles were different across ages. We also performed a measurement invariance test across informants at age 3. We compared the model for mother reports with a model with also four profiles for father reports at age 3. The measurement invariance test favored a model with equal profiles across informants (BIC = 240,900) above a model with different profiles across informants (BIC = 241,011).

**Fig. 1:**
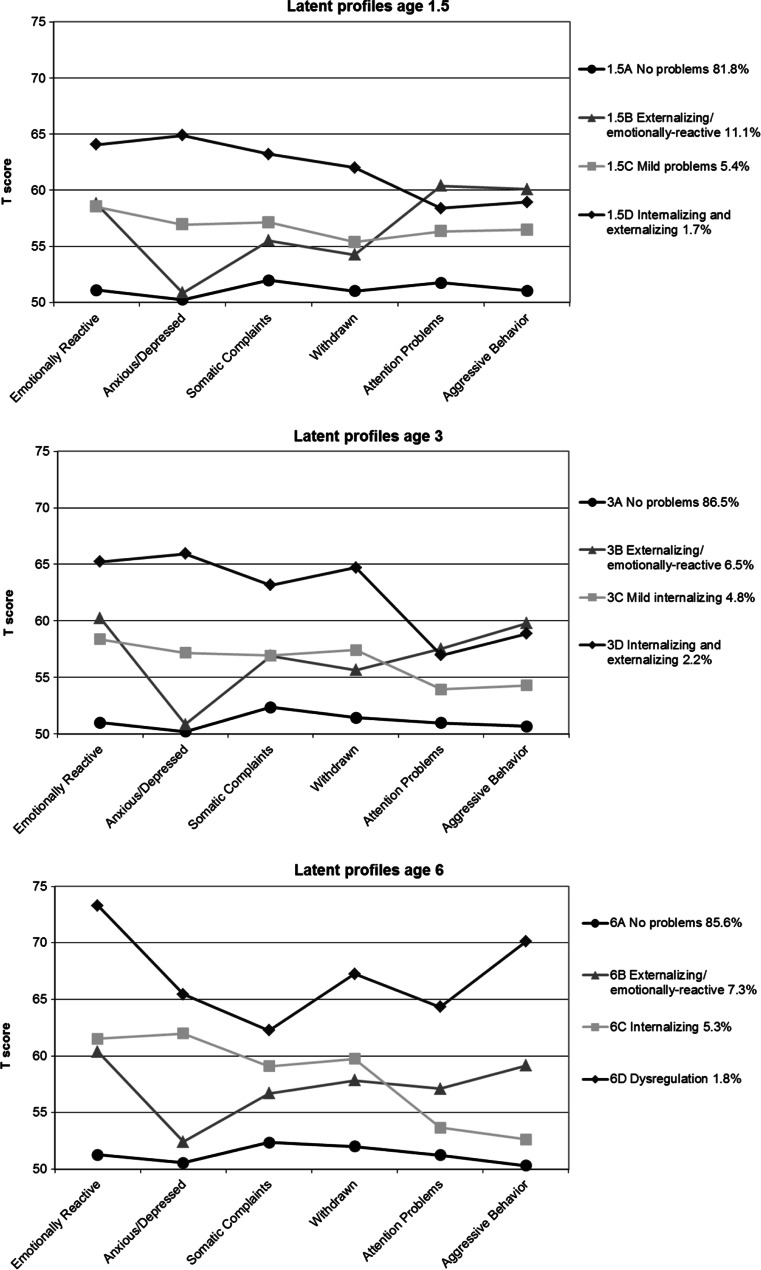
Mean T-scores for latent profile models at ages 1.5, 3 and 6 years. Note Latent profile model at age 6 adapted from (Basten et al. 2013)

Figure 1 shows the profiles at ages 1.5, 3, and 6. At each age most children were in a profile without problems: profile 1.5A (81.8 %), profile 3A (86.5 %), and profile 6A (85.6 %). These profiles were labeled ‘No problems’. Additionally, at each age there was a profile with moderate externalizing problems and emotionally-reactive behavior and low levels of anxiety and depression: profiles 1.5B, 3B, and 6B. We labeled these profiles ‘Externalizing/emotionally-reactive’. The prevalence of these profiles was higher at age 1.5 (11.1 %) than at ages 3 (6.5 %) and 6 (7.3 %). The ‘Internalizing’ profile at age 6 (6C; 5.3 %), with moderate scores on all internalizing scales and no elevations on the externalizing scales, was not observed at earlier ages. Instead, at age 3 there was a profile (3C; 4.8 %) with only slightly higher scores on the internalizing scales than on the externalizing scales. This profile was labeled ‘Mild internalizing’. At age 1.5 we found a profile (1.5C; 5.4 %) with mild problems on all scales and was therefore labeled ‘Mild problems’. Finally, at each age there was a profile with moderate to high scores across all internalizing and externalizing scales: profile 1.5D (1.7 %), profile 3D (2.2 %), and profile 6D ‘Dysregulation’ (1.8 %). Profiles 1.5D and 3D differed from the ‘Dysregulation’ profile at age 6 in that they scored lower on Emotionally Reactive, Attention Problems, and Aggressive Behavior. Profiles 1.5D and 3D were labeled ‘Internalizing and externalizing’.

### Transitions Over Time

We performed latent transition analysis to examine the stability of problem behavior in children. The transition probabilities represent the likelihood to move from a certain profile at ages 1.5 or 3 to another profile at a later age. Table 2 shows the transition probabilities from age 1.5 to age 3 and from ages 1.5 and 3 to age 6. Children in the ‘No problems’ profiles at age 1.5 (probability 0.89) and at age 3 (probability 0.92) were very likely to be in the ‘No problems’ profile at age 6. In contrast, children in the ‘Internalizing and externalizing’ profiles at ages 1.5 (probability 0.57) and 3 (probability 0.32) were less likely to be in the ‘No problems’ profile at age 6. In other words, the probabilities for children with combined internalizing and externalizing problems at age 1.5 or 3 to be in one of the three problem profiles at age 6 were 0.43 and 0.68 respectively.

**Table 2:** Transition probabilities from age 1.5 to age 3, and from ages 1.5 and 3 to age 6 (*N* = 7,206)

	Profiles age 3			
	3A No problems (*n* = 6,070)	3B Externalizing/emotionally-reactive (*n* = 566)	3C Mild internalizing (*n* = 363)	3D Internalizing and externalizing (*n* = 178)
Profiles age 1.5				
1.5A No problems (*n* = 5,815)	0.93	0.04	0.03	0.01
1.5B Externalizing/emotionally-reactive (*n* = 847)	0.52	0.31	0.11	0.05
1.5C Mild problems (*n* = 407)	0.51	0.16	0.22	0.12
1.5D Internalizing and externalizing (*n* = 137)	0.31	0.15	0.17	0.37
	Profiles age 6			
	6A No problems (*n* = 6,114)	6B Externalizing/emotionally-reactive (*n* = 560)	6C Internalizing (*n* = 393)	6D Dysregulation (*n* = 140)
Profiles age 1.5				
1.5A No problems (*n* = 5,815)	0.89	0.06	0.04	0.01
1.5B Externalizing/emotionally-reactive (*n* = 847)	0.70	0.17	0.09	0.05
1.5C Mild problems (*n* = 407)	0.70	0.13	0.12	0.05
1.5D Internalizing and externalizing (*n* = 137)	0.57	0.15	0.19	0.09
	Profiles age 6			
	6A No problems (*n* = 6,114)	6B Externalizing/emotionally-reactive (*n* = 560)	6C Internalizing (*n* = 393)	6D Dysregulation (*n* = 140)
Profiles age 3				
3A No problems (*n* = 6,070)	0.92	0.04	0.03	0.01
3B Externalizing/emotionally-reactive (*n* = 566)	0.43	0.39	0.08	0.09
3C Mild internalizing (*n* = 363)	0.60	0.12	0.23	0.05
3D Internalizing and externalizing (*n* = 178)	0.32	0.15	0.35	0.18

Profile sizes represent profile counts based on estimated model

Transition probabilities to move from one of the three problem profiles at age 1.5 to any specific problem profile at age 6 were all below 0.20, whereas these probabilities from age 3 to age 6 go up to 0.39 for the profiles ‘Externalizing/emotionally-reactive’. LTA in complete cases yielded almost identical transition probabilities (data not shown).

We also examined transitions conversely by investigating the previous profile membership for those children who were in one of the three problem profiles at age 6. To this aim, we calculated profile membership probabilities at ages 1.5 and 3 conditional on profile membership at age 6 (probabilities are shown in Supplementary Table S2). Children in the ‘Dysregulation’ profile at age 6 were most likely to have had any problems at younger ages. These probabilities were 0.72 (age 3) and 0.52 (age 1.5). In comparison, for children in the ‘Externalizing/emotionally-reactive’ profile and ‘Internalizing’ profile at age 6 the probabilities to have had any problems at age 3 were 0.52 and 0.49, respectively.

### Gender and SES Differences

Boys were more likely to be in the ‘Dysregulation’ profile at age 6 (OR = 2.04, *p* < 0.001) than girls, while there was no relation between gender and the ‘Internalizing and externalizing’ profiles at age 1.5 (OR = 1.14, *p* = 0.539) and age 3 (OR = 1.26, *p* = 0.237). Boys were also more likely to be in the ‘Externalizing/emotionally-reactive’ at age 6 (OR = 1.86, *p* < 0.001) and age 3 (OR = 1.52, *p* < 0.001), but not at age 1.5 (OR = 1.11, *p* = 0.232). Gender was not related to the other profiles. We added gender to the LTA model to examine transition probabilities per gender (gender specific probabilities are shown in Supplementary Table S3). Most transition probabilities differed only slightly by gender. Boys in the profile ‘Externalizing/emotionally-reactive’ at age 3 (probability 0.44) appeared more likely than girls (probability 0.32) to move again to the ‘Externalizing/emotionally-reactive’ profile at age 6.

Children from mothers with a medium/low education level were more likely to be in the following problem profiles: age 1.5 ‘Internalizing and externalizing’ (OR = 2.80, *p* < 0.001) and ‘Mild problems’ (OR = 1.91, *p* < 0.001), age 3 ‘Mild internalizing’ (OR = 1.67, *p* < 0.001), age 6 ‘Dysregulation’ (OR = 2.07, *p* < 0.001) and ‘Internalizing’ (OR = 1.62, *p* < 0.001). Looking at transition probabilities for children from medium/low educated mothers and highly educated mothers we found small differences (See Supplementary Table S4). These findings were very similar to those of family income (data not shown).

## Discussion

This study examined the stability of internalizing problems, externalizing problems, and their co-occurrence from age 1.5 to 6 years, while taking into account developmental changes in the presentation of problems. Using LPA, we showed that the presentation of internalizing and externalizing problems changed from 1.5 to 3 and to 6 years. Most notably were changes in the presentation of co-occurring internalizing and externalizing problems over time: a profile with co-occurring internalizing and externalizing problems was found at all ages but was characterized by more severe problems at 6 years than at 1.5 and 3 years. A profile with predominantly internalizing problems was only discernible at 6 years, while at earlier ages internalizing problems were accompanied by at least mild levels of externalizing problems. In contrast, a profile characterized by moderate externalizing problems and emotionally-reactive behavior was visible at all ages.

To our knowledge, this is the first study that used LTA to empirically identify profiles of internalizing and externalizing problems and their co-occurrence at different stages of the preschool period and to examine the stability of problems in a large general population sample. The lack of measurement invariance in profiles across ages in our study suggests that children are very likely to show different patterns of problems across the preschool period. This phenomenon that we called heterotypic stability was further supported by the results from the LTA analysis. Furthermore, transition probabilities showed that, although children with problems at ages 1.5 and 3 were at increased risk to show problems again at 6 years, it was difficult to predict what kind of problem profile a child would exhibit at 6 years. The transition probabilities of having any problems across ages are highly similar to overall stability rates from previous studies (Briggs-Gowan et al. 2006; Bufferd et al. 2012; Lavigne et al. 1998). For example, we found a 40 to 68 % stability of any problems from 3 to 6 years which was comparable to a stability of 50 % of having any psychiatric disorder across this same age span reported by Bufferd et al. (2012) using a categorical approach. Changes in the presentation of problems in children with persistent problems have also been identified by others (Briggs-Gowan et al. 2006; Bufferd et al. 2012; Lavigne et al. 1998; Mesman et al. 2001). Bufferd et al. (2012) found that the likelihood to have the same DSM diagnosis from 3 to 6 years of age was equal to the likelihood to meet the criteria for a different DSM diagnosis. By using an empirical approach to define profiles of internalizing and externalizing problems, we found that heterotypic stability in the general population is even more common than expected based on previous studies. These findings have critical implications for future studies on the continuity of problems but also in the investigation of treatment effects. Examination of long term outcomes of a particular “disorder” need to be examining psychopathology broadly, as developmental effects will change the presentation of problem behavior.

The presentation of different types of problems over time may be the result of different causes. An alternative explanation for heterotypic stability is an underlying syndrome of poor self-regulation that may result in both internalizing and externalizing problems. Self-regulation is in young children also described as effortful control or emotion self-regulation (Eisenberg et al. 2010). Children with deficits in emotion self-regulation have difficulties in inhibiting their behavior when emotionally aroused, which may result in externalizing problems. Children with deficits in emotion self-regulation may also have more difficulties in inhibiting negative thoughts and in shifting attention from negative stimuli resulting in internalizing problems (Eisenberg et al. 2010). The fact that children in the Internalizing, the Externalizing/emotionally-reactive, and the Dysregulation profile at 6 years have high scores on the Emotionally Reactive scale of the preschool CBCL may reflect this underlying syndrome of poor self-regulation.

At all ages a profile with moderate to high levels of co-occurring internalizing and externalizing problems was identified, but at 6 years this profile was characterized by more severe problems on the Emotionally Reactive, Attention Problems and Aggressive Behavior scales. From the viewpoint that co-occurrence of internalizing and externalizing problems stems from an underlying syndrome of poor self-regulation, we previously labeled this profile at 6 years ‘Dysregulation’ (Basten et al. 2013). A possible explanation for the finding that the profile shows higher scores in 6 year-olds is that by this age children have entered school where self-regulatory skills are required. Problems in regulating emotions, attention and behavior might become more impairing and more visible for the environment at this age (see also Blair 2002). The finding of a highly dysregulated group only at 6 years appears also in line with studies showing higher prevalence of comorbid DSM diagnoses at the end of the preschool period (Egger and Angold 2006; Lavigne et al. 1998).

Another finding related to co-occurrence was that latent profiles at 1.5 and 3 years showed that internalizing problems were accompanied by at least mild forms of externalizing problems. A possible explanation for the absence of a profile with predominantly internalizing problems at these ages is that young children have limited ability to communicate about their emotions and might use also externalizing behavior to express their feelings (Gardner and Shaw 2008). Also, internalizing problems, such as separation anxiety, might become more impairing when children have to go to school, while at younger ages these problems are seen as developmentally normal emotions (Gardner and Shaw 2008). Based on factor analytical studies showing the distinction between internalizing and externalizing problems at early age (Achenbach and Rescorla 2000; Carter et al. 2003), it is often assumed that children with only internalizing problems can be identified. However, based on our results, we suggest that future studies should take into account that 1.5- to 3-year-old children with parent reported internalizing problems are also likely to have at least mild levels of externalizing problems.

Although much of the findings suggested heterotypic stability, there were some findings supporting stability of the same pattern of problems over time. The profile Externalizing/emotionally-reactive showed very similar profiles across ages. Furthermore, children in this profile at age 3 were more likely to move again to this profile at age 6 than moving to another problem profile. These findings are in line with a higher homotypic stability found for externalizing problems than for internalizing problems by others (Briggs-Gowan et al. 2006; Fischer et al. 1984). The prevalence of this class was higher at 1.5 years than at older ages. This finding is in agreement with existing literature reporting that externalizing behavior is more common at early age (Gardner and Shaw 2008).

Stability of any problems was highest for children with moderate to high levels of co-occurring internalizing and externalizing problems, with almost 70 % of children in a co-occurring profile at age 3 having again problems at age 6. This has been previously found in preschool children (Briggs-Gowan et al. 2006). Studies in school-age children have shown that co-occurrence of internalizing and externalizing problems is a very strong risk factor for adult psychopathology (Althoff et al. 2010; Sourander et al. 2007). These findings suggest that prevention and intervention strategies should target those children with co-occurring internalizing and externalizing problems to prevent them from developing severe psychopathology later in life. In addition, other risk factors that play a role in the stability of problems should be considered in the development of intervention strategies. Several biological and environmental factors have been found to predict stability of problems over time, such as poor family functioning, stressful life events and physical health problems (Briggs-Gowan et al. 2006; Campbell et al. 2000; Mesman and Koot 2001). Including these factors, as well as levels of impairment, may improve the identification of children at risk for persistent problems.

This study demonstrated a gradual emergence of a higher prevalence of boys in profiles characterized by externalizing problems and a higher stability of externalizing problems in boys, which is in line with existing literature (Hay 2007; Rutter et al. 2003). Previous studies on categorical stability did not find these gender differences (Briggs-Gowan et al. 2006; Bufferd et al. 2012; Lavigne et al. 1998), which might be related to power limitations. Hay (2007) proposes that these emerging differences are most likely related to earlier maturation of girls, boys’ vulnerability, and differences in social influences.

Strengths of the current study were the large number of children, the population-based design and the use of multiple informants when the child was 3 years old. Also, children were classified empirically and not on arbitrarily chosen cut-points. There were also limitations. The LTA only comprised mother reports collected at ages 1.5, 3, and 6. We could not conduct an LTA with data of other informants. However, we collected father reports at age 3. A test comparing profiles between mothers and fathers at age 3 showed that these profiles were invariant across informants. In addition, our study only relied on questionnaires completed by parents, but the use of data collected from other informants such as caregivers in preschool or daycare centers or the use of observational data would further advance knowledge of the development of problem behavior in children. The internal consistency of the Somatic Complaints and Withdrawn scales was poor (below 0.70 at all ages). These scales did not play an important role in the distinction between profiles. A likely explanation is that these types of problems are often secondary to symptoms of anxiety, aggressive behavior and attention problems. Also, for the decision on the number of profiles in LPA we used statistical and substantive criteria in agreement with other studies (Collins and Lanza 2010; Meeus et al. 2011). At age 1.5 a fifth profile emerged that was similar in shape to, but with somewhat higher scores than the already existing profile Externalizing/Emotionally reactive. Because of this resemblance we selected the four-profile solution instead of the five-profile solution. Another limitation is that at “age 6” children were actually 5-to-7 years old. To examine whether profiles were influenced by age differences at this wave, we added age as a covariate in the LPA (Basten et al. 2013). Model fit did not improve, suggesting that age differences had little effect on profiles. Also, the participation rate in the present study was high (74 %) but the possibility of selection bias remains, as the non-respondents had more often a lower socioeconomic status. This limits the generalizability of the results to children with low socio-economic background. We found that children with a lower economic background were more likely to be in a problem profile at each age. Therefore the prevalence of problem behavior might have been higher in the non-response group. Finally, this study examined whether the stability of problem behavior depends on gender and socioeconomic background. In future research we recommend to also add other covariates to the latent transition models to see whether transitions in time are influenced by factors such as parental psychopathology and parenting styles.

In conclusion, we showed that the presentation of internalizing and externalizing problems in the general population changes during the preschool period. In addition, this study showed that heterotypic continuity of problems is very common among preschoolers, and that children with high levels of co-occurring internalizing and externalizing problems are most likely to have persisting problems. The identified profiles in the current study do not represent clinical diagnoses. However, our findings have several implications for clinical practice and research. First, when examining the stability of a child’s problems over time or studying the effects of treatment, the whole range of psychopathology should be considered with age appropriate instruments as the presentation of problems likely has changed. Second, children with co-occurring problems deserve early intervention. The treatment of children with co-occurring internalizing and externalizing problems may need to be of a longer duration and may need to attend to both types of problems. A family-based intervention in preschoolers has been found to be especially beneficial to children with co-occurring problems (Connell et al. 2008). Lastly, the co-occurrence of problems as well as the high levels of heterotypic stability may reflect one underlying syndrome of poor self-regulation. A common factor underlying internalizing and externalizing problems has been recognized by many (e.g., Eisenberg et al. 2010; Mikita and Stringaris 2013). Studying this factor may help improve our understanding of problem behavior across development.

## Electronic Supplementary Material

Below is the link to the electronic supplementary material.

Supplemental Table 1 (DOCX 35.4 kb)

Supplemental Table 2 (DOCX 43 kb)

Supplemental Table 3 (DOCX 38.0 kb)

Supplemental Table 4 (DOCX 38.1 kb)
