# Supplementary material for: The Stability of Problem Behavior Across the Preschool Years: An Empirical Approach in the General Population
Source: J Abnorm Child Psychol. 2015 Apr 2;44(2):393–404. doi: 10.1007/s10802-015-9993-y (PMC4729812; doi:10.1007/s10802-015-9993-y)
Supplement: Supplementary file 1 — (DOCX 35.4 kb) [file 10802_2015_9993_MOESM1_ESM.docx]

**Supplementary table S1** Fit statistics for latent profile models at ages 1.5, 3, and 6 years

|  | Number of Profiles | BIC | BLRT | Entropy | Smallest profile |
| --- | --- | --- | --- | --- | --- |
| Age 1.5 (n=5,182) | 1 | 176,330 | - | - | - |
|  | 2 | 167,397 | <0.001 | 0.96 | 13.2% |
|  | 3 | 163,105 | <0.001 | 0.96 | 4.3% |
|  | 4 | 158,942 | <0.001 | 0.97 | 1.7% |
|  | 5 | 157,784 | <0.001 | 0.98 | 1.7% |
| Age 3 (n=4,928) | 1 | 165,393 | - | - | - |
|  | 2 | 155,495 | <0.001 | 0.98 | 8.7% |
|  | 3 | 151,292 | <0.001 | 0.98 | 2.5% |
|  | 4 | 147,230 | <0.001 | 0.99 | 2.2% |
|  | 5 | 145,126 | <0.001 | 0.99 | 0.6% |
| Age 6* (n=6,131) | 1 | 217,304 | - | - | - |
|  | 2 | 204,285 | <0.001 | 0.98 | 8.9% |
|  | 3 | 199,746 | <0.001 | 0.97 | 2.8% |
|  | 4 | 196,759 | <0.001 | 0.98 | 1.8% |
|  | 5 | 194,392 | <0.001 | 0.99 | 0.9% |

* Results were previously published in Basten et al. (2013).

BIC = Bayesian information criterion, BLRT = Bootstrapped likelihood-ratio test.
