# Supplementary material for: The Stability of Problem Behavior Across the Preschool Years: An Empirical Approach in the General Population
Source: J Abnorm Child Psychol. 2015 Apr 2;44(2):393–404. doi: 10.1007/s10802-015-9993-y (PMC4729812; doi:10.1007/s10802-015-9993-y)
Supplement: Supplementary file 2 — (DOCX 43 kb) [file 10802_2015_9993_MOESM2_ESM.docx]

**Supplementary table S2** Profile membership probabilities at ages 1.5 and 3 conditional on profile membership at age 6 (N=7,206).

|  | **Profiles age 1.5** | | | |
| --- | --- | --- | --- | --- |
|  | 1.5A No problems  (n=5,815) | 1.5B Externalizing/emotionally-reactive (n=847) | 1.5C Mild problems  (n=407) | 1.5D Internalizing and externalizing (n=137) |
| **Profiles age 6** |  |  |  |  |
| 6A No Problems (n=6,114) | 0.84 | 0.10 | 0.05 | 0.01 |
| 6B Externalizing/emotionally-reactive (n=560) | 0.62 | 0.25 | 0.09 | 0.04 |
| 6C Internalizing (n=393) | 0.62 | 0.19 | 0.13 | 0.07 |
| 6D Dysregulation (n=140) | 0.48 | 0.29 | 0.14 | 0.09 |
|  | **Profiles age 3** | | | |
|  | 3A No problems  (n=6,070) | 3B Externalizing/emotionally-reactive (n=566) | 3C Mild internalizing  (n=363) | 3D Internalizing and externalizing (n=178) |
| **Profiles age 6** |  |  |  |  |
| 6A No Problems (n=6,114) | 0.92 | 0.04 | 0.04 | 0.01 |
| 6B Externalizing/emotionally-reactive (n=560) | 0.48 | 0.40 | 0.08 | 0.05 |
| 6C Internalizing (n=393) | 0.51 | 0.12 | 0.21 | 0.16 |
| 6D Dysregulation (n=140) | 0.28 | 0.37 | 0.12 | 0.23 |

Note. Profile sizes represent profile counts based on estimated model.
