# Supplementary material for: The Stability of Problem Behavior Across the Preschool Years: An Empirical Approach in the General Population
Source: J Abnorm Child Psychol. 2015 Apr 2;44(2):393–404. doi: 10.1007/s10802-015-9993-y (PMC4729812; doi:10.1007/s10802-015-9993-y)
Supplement: Supplementary file 3 — (DOCX 38.0 kb) [file 10802_2015_9993_MOESM3_ESM.docx]

**Supplementary table S3** Transition probabilities from age 1.5 to age 3 and from ages 1.5 and 3 to age 6 for boys and girls separately.

|  | | **Profiles age 3** | | | |
| --- | --- | --- | --- | --- | --- |
|  | | 3A No problems | 3B Externalizing/emotionally-reactive | 3C Mild internalizing | 3D Internalizing and externalizing |
| **Profiles age 1.5** | | boys / girls | boys / girls | boys / girls | boys / girls |
| 1.5A No problems | | 0.92 / 0.94 | 0.04 / 0.03 | 0.03 / 0.03 | 0.01 / 0.00 |
| 1.5B Externalizing/emotionally-reactive | | 0.49 / 0.55 | 0.36 / 0.26 | 0.11 / 0.12 | 0.04 / 0.07 |
| 1.5C Mild problems | | 0.52 / 0.50 | 0.18 / 0.13 | 0.17 / 0.27 | 0.13 / 0.10 |
| 1.5D Internalizing and externalizing | | 0.33 / 0.30 | 0.16 / 0.13 | 0.14 / 0.21 | 0.37 / 0.37 |
|  |  | **Profiles age 6** | | | |
|  |  | 6A No problems | 6B Externalizing/emotionally-reactive | 6C Internalizing | 6D Dysregulation |
| **Profiles age 1.5** | | boys / girls | boys / girls | boys / girls | boys / girls |
| 1.5A No problems | | 0.86 / 0.91 | 0.08 / 0.04 | 0.04 / 0.04 | 0.02 / 0.01 |
| 1.5B Externalizing/emotionally-reactive | | 0.66 / 0.73 | 0.21 / 0.12 | 0.08 / 0.11 | 0.05 / 0.04 |
| 1.5C Mild problems | | 0.69 / 0.72 | 0.16 / 0.10 | 0.10 / 0.14 | 0.06 / 0.05 |
| 1.5D Internalizing and externalizing | | 0.56 / 0.57 | 0.18 / 0.11 | 0.16 / 0.22 | 0.10 / 0.10 |
|  |  | **Profiles age 6** | | | |
|  |  | 6A No problems | 6B Externalizing/emotionally-reactive | 6C Internalizing | 6D Dysregulation |
| **Profiles age 3** | | boys / girls | boys / girls | boys / girls | boys / girls |
| 3A No problems | | 0.90 / 0.94 | 0.06 / 0.03 | 0.03 / 0.03 | 0.01 / 0.00 |
| 3B Externalizing/emotionally-reactive | | 0.39 / 0.50 | 0.44 / 0.32 | 0.07 / 0.10 | 0.10 / 0.08 |
| 3C Mild internalizing | | 0.63 / 0.58 | 0.14 / 0.10 | 0.19 / 0.27 | 0.05 / 0.05 |
| 3D Internalizing and externalizing | | 0.34 / 0.30 | 0.18 / 0.11 | 0.31 / 0.39 | 0.18 / 0.20 |
