# Supplementary material for: The Stability of Problem Behavior Across the Preschool Years: An Empirical Approach in the General Population
Source: J Abnorm Child Psychol. 2015 Apr 2;44(2):393–404. doi: 10.1007/s10802-015-9993-y (PMC4729812; doi:10.1007/s10802-015-9993-y)
Supplement: Supplementary file 4 — (DOCX 38.1 kb) [file 10802_2015_9993_MOESM4_ESM.docx]

**Supplementary table S4** Transition probabilities from age 1.5 to age 3 and from ages 1.5 and 3 to age 6 for children of low-medium educated mothers and children of high educated mothers separately.

|  | | **Profiles age 3** | | | |
| --- | --- | --- | --- | --- | --- |
|  | | 3A No problems | 3B Externalizing/emotionally-reactive | 3C Mild internalizing | 3D Internalizing and externalizing |
| **Profiles age 1.5** | | low-medium/high | low-medium/high | low-medium/high | low-medium/high |
| 1.5A No problems | | 0.91 / 0.94 | 0.04 / 0.03 | 0.04 / 0.02 | 0.00 / 0.00 |
| 1.5B Externalizing/emotionally-reactive | | 0.53 / 0.56 | 0.31 / 0.31 | 0.14 / 0.07 | 0.03 / 0.06 |
| 1.5C Mild problems | | 0.47 / 0.61 | 0.16 / 0.16 | 0.23 / 0.16 | 0.13 / 0.07 |
| 1.5D Internalizing and externalizing | | 0.31 / 0.23 | 0.13 / 0.27 | 0.22 / 0.15 | 0.35 / 0.35 |
|  |  | **Profiles age 6** | | | |
|  |  | 6A No problems | 6B Externalizing/emotionally-reactive | 6C Internalizing | 6D Dysregulation |
| **Profiles age 1.5** | | low-medium/high | low-medium/high | low-medium/high | low-medium/high |
| 1.5A No problems | | 0.87 / 0.91 | 0.06 / 0.06 | 0.05 / 0.03 | 0.02 / 0.01 |
| 1.5B Externalizing/emotionally-reactive | | 0.70 / 0.71 | 0.16 / 0.18 | 0.09 / 0.08 | 0.05 / 0.04 |
| 1.5C Mild problems | | 0.67 / 0.76 | 0.14 / 0.11 | 0.13 / 0.08 | 0.06 / 0.04 |
| 1.5D Internalizing and externalizing | | 0.56 / 0.54 | 0.16 / 0.19 | 0.18 / 0.19 | 0.10 / 0.08 |
|  |  | **Profiles age 6** | | | |
|  |  | 6A No problems | 6B Externalizing/emotionally-reactive | 6C Internalizing | 6D Dysregulation |
| **Profiles age 3** | | low-medium/high | low-medium/high | low-medium/high | low-medium/high |
| 3A No problems | | 0.90 / 0.93 | 0.04 / 0.04 | 0.04 / 0.03 | 0.01 / 0.00 |
| 3B Externalizing/emotionally-reactive | | 0.46 / 0.39 | 0.35 / 0.44 | 0.06 / 0.09 | 0.12 / 0.07 |
| 3C Mild internalizing | | 0.57 / 0.69 | 0.14 / 0.06 | 0.26 / 0.20 | 0.03 / 0.04 |
| 3D Internalizing and externalizing | | 0.27 / 0.34 | 0.21 / 0.14 | 0.31 / 0.34 | 0.22 / 0.18 |

Note: Maternal education was defined as highest education finished. Education categories represent low-medium: primary school, lower vocational education or intermediate vocational education; high: higher vocational education or university.
